# Ketogenic Diet and Weight Loss: Is There an Effect on Energy Expenditure?

**DOI:** 10.3390/nu14091814

**Published:** 2022-04-26

**Authors:** Alessio Basolo, Silvia Magno, Ferruccio Santini, Giovanni Ceccarini

**Affiliations:** Obesity and Lipodystrophy Center, Endocrinology Unit, University Hospital of Pisa, 56124 Pisa, Italy; alessio.basolo@med.unipi.it (A.B.); silviamagno9@gmail.com (S.M.); ferruccio.santini@unipi.it (F.S.)

**Keywords:** ketogenic diet, energy expenditure, food intake, thermic effect of food

## Abstract

A dysregulation between energy intake (EI) and energy expenditure (EE), the two components of the energy balance equation, is one of the mechanisms responsible for the development of obesity. Conservation of energy equilibrium is deemed a dynamic process and alterations of one component (energy intake or energy expenditure) lead to biological and/or behavioral compensatory changes in the counterpart. The interplay between energy demand and caloric intake appears designed to guarantee an adequate fuel supply in variable life contexts. In the past decades, researchers focused their attention on finding efficient strategies to fight the obesity pandemic. The ketogenic or “keto” diet (KD) gained substantial consideration as a potential weight-loss strategy, whereby the concentration of blood ketones (acetoacetate, 3-β-hydroxybutyrate, and acetone) increases as a result of increased fatty acid breakdown and the activity of ketogenic enzymes. It has been hypothesized that during the first phase of KDs when glucose utilization is still prevalent, an increase in EE may occur, due to increased hepatic oxygen consumption for gluconeogenesis and for triglyceride-fatty acid recycling. Later, a decrease in 24-h EE may ensue due to the slowing of gluconeogenesis and increase in fatty acid oxidation, with a reduction of the respiratory quotient and possibly the direct action of additional hormonal signals.

## 1. Introduction

Obesity is a relevant public health issue, and its prevalence has increased dramatically in the past decades. It is estimated that 39% of the globe population is overweight and 13% suffer from obesity [1]. The chronic uncoupling of the two components of the energy balance equation, energy intake (EI) and energy expenditure (EE), is classically thought to be responsible for the progressive increase in body weight [2,3,4]. Daily caloric intake oscillates due to various individual and environmental factors. On the other hand, energy expenditure is influenced by body composition, physical activity, and food utilization [5]. Although a “lifestyle change approach”, aimed at reducing caloric intake and increasing physical activity, is crucial to promote weight loss, there is no strong evidence to recommend one dietary regimen as more effective over another. For the theory based on the “carbohydrate–insulin model”, a relative increase in carbohydrate intake leads to an increase in insulin secretion that facilitates the accumulation of body fat [6,7]. This model has been disputed [8] since it claims the exclusivity of the insulin and carbohydrate role in the development of obesity. Indeed, adipose fat storage might also occur during low-carbohydrate dietary regimens whenever energy consumption exceeds expenditure. Variations of leptin and incretin levels, amino acid intake, and the autonomic neural system activity all converge on the central mechanisms of control of energy balance and may favor an increased body adiposity, independently of carbohydrate intake or insulin levels [9,10,11,12]. In any case, the physiological premise of ketogenic diets (KDs) is that by reducing carbohydrate intake, without altering the protein load, a decrease in insulin secretion will follow, promoting fatty acid oxidation. Fat mobilization will occur in case a caloric deficit is established, which is facilitated by the anorectic effect of ketone bodies, with minor effects on lean body mass. Whether KDs affect energy expenditure is poorly understood and, to date, very few studies have evaluated the impact of KDs on energy consumption. The aim of the current review was to describe the current evidence on the potential effect of KDs on energy expenditure in subjects with obesity.

## 2. The Ketogenic Diet

KDs are characterized by a markedly reduced carbohydrate intake (<30 g/day) and a normal protein contribution (1.2–1.5 g/kg of ideal body weight or 1.0–1.2 g/kg of fat free mass). They are frequently used as therapeutic strategies in the treatment of obesity, type 2 diabetes mellitus, and other medical conditions, such as migraine [13,14,15], polycystic ovary syndrome [16], cancer [17], neurodegenerative diseases [18,19,20], and epilepsy [21]. Furthermore, it has been proposed that ketone bodies might play a role in the regulation of biological processes (meal timing, appetite, and sleep cycle) involved in the maintenance of circadian rhythms (elegantly reviewed in [22]), the desynchronization of which might lead to development of obesity [4]. A reduction of mitochondrial function leading to the excessive production of reactive oxygen species appears to be involved in the etiology of several chronic diseases, including obesity and diabetes [23]. It has been hypothesized that KDs may stimulate the mitochondrial function and the endogenous antioxidant defense, by increasing hormones (i.e., adiponectin) that activate key enzymes involved in the induction of signal transduction, including AMP-activated protein kinase (AMPK) [24]. KDs gained substantial consideration in 1970s when they were advertised and commercialized by Dr. Atkins as an efficacious weight-loss strategy [25]. The most used variants of KDs in obesity management can be distinguished in two types, based on calories introduced: (1) LCKD: low calorie ketogenic diet, providing a total daily energy intake of 800–1200 [26,27]; (2) VLCKD: very low calorie ketogenic diet, with an energy intake of <800 kcal/day [28,29,30].

**Figure 1:**
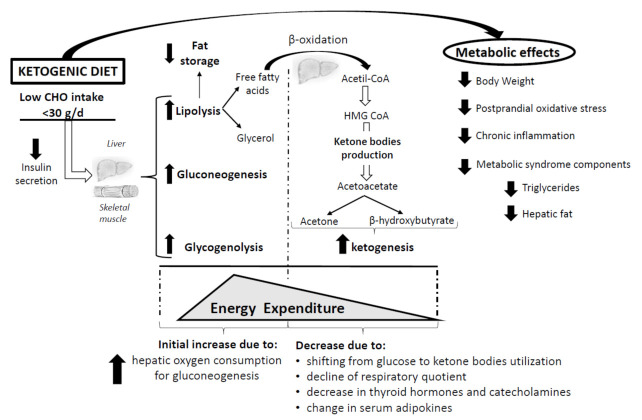
Metabolic effects of ketogenic diets. Carbohydrates are the primary source of energy production in body tissues. When carbohydrate intake is less than 30 g per day, insulin secretion decreases, leading to an increase in glycogenolysis, gluconeogenesis, and lipolysis. When glucose disposal decreases further, gluconeogenesis cannot replace the necessary needs of the body. Thus, the increased lipolysis leads to the enhanced production of free fatty acids which, in turn, are converted to Acetil-CoA through beta-oxidation. This process leads to the production of ketone bodies (acetoacetate, acetone, and 3-β-hydroxybutyrate) that provide “extra fuel” to various tissues. The beneficial metabolic effects of the KDs are represented on the right side of Table 1. Regarding the effects on energy metabolism, it has been hypothesized that a rapid increase in EE may initially occur due to increased hepatic oxygen consumption for gluconeogenesis. Later, a decrease in 24-h EE occurs due to the slowing of gluconeogenesis, a decrease in the respiratory quotient due to increased utilization of ketone bodies, a reduction of thyroid hormones and catecholamines, and changes in serum adipokines. g/d: grams per day.

The reduction of insulin concentration, caused by a low carbohydrate intake, leads to metabolic changes, such as increased glycogenolysis, gluconeogenesis, and lipolysis [31,32,33,34,35]. When the stored glucose is fully depleted, the body starts to use fat as the primary energetic substrate. The latter catabolic pathway is responsible for the production of free fatty acids which, in turn, are broken down to acetyl coenzyme A (CoA) through beta-oxidation in the mitochondria of hepatocytes. Acetyl coenzyme A (CoA) is converted to 3-hydroxy-3-methyl-glutaryl-CoA (HMG CoA) which, in turn, generates the ketone body acetoacetate [36], starting the ketogenic process. Further, acetoacetate can be converted to acetone or 3-β-hydroxybutyrate [36]. Ketone bodies are an alternative energetic substrate generated from stored fat for specific organs, including the heart, brain, and skeletal muscle [31,36,37,38] (Figure 1). The metabolic state of ketosis observed during fasting or KD is benign and should not be confused with the pathological ketoacidosis occurring in type I diabetes mellitus [32], a harmful condition [39].

## 3. Hints on Energy Expenditure Components

The rate of whole-body energy expenditure (EE) changes significantly over the 24-h period and can be precisely measured with an open circuit whole room indirect calorimeter, also known as the respiratory chamber [40,41]. This method is considered the gold standard for the measurement of energy expenditure over 24 h because of the ability to differentiate its components, such as resting metabolic rate (RMR), thermogenic effect of food, and the energy cost of physical activity.

Lean body mass is the main determinant of RMR, accounting for ~70% of its variance [5,42,43], while other factors, such as fat mass, sex, age, race, and familial genetic background, explain an extra ~15% [44]. The raise in energy needed to digest and absorb nutrients following food consumption [45] is defined as thermogenic effect of food and it accounts for ~10% of the 24-h EE [46,47], although a large variability among subjects can be observed. The energy consumed for spontaneous and voluntary physical activity has proven the most flexible element of 24-h EE, diverging from ~15% in sedentary individuals to ~50% in active subjects [48].

## 4. Energy Balance Regulation and Metabolic Adaption

Historically, the mechanisms underlying the pathogenesis of obesity are based on a simplistic model of energy balance, which implies that a chronic positive energy balance (i.e., caloric intake constantly overcoming energy expenditure) causes a storage of energy surplus with a consequent increase in body weight [2]. This static model presumes that oscillations in energy intake and energy expenditure affect the energy balance in an independent manner. Emerging evidence suggests that these components are controlled by a more complex model in which the perturbation of one component (energy intake or energy expenditure) leads to biological and/or behavioral compensatory changes in the other, with the aim of preserving body weight and body energy stores within a steady range. Specifically, an increase in the energy expenditure would lead to increased food intake (and vice versa), whereas a decrease in the energy demand would lead to reduction of the energy intake (and vice versa) [3,49,50]. Recently, researchers have focused their attention on studying the adaptive response of one component to changes of the other constituent of the energy balance equation. These studies have led to the identification of different metabolic phenotypes (“spendthrift” and “thrifty”) that are classified based on changes in EE during acute (fasting or overfeeding) dietary interventions [51,52,53,54]. The “spendthrift” phenotype is characterized by smaller decreases in EE during fasting and larger increases in EE during overfeeding, whereas the “thrifty” one is an energy-efficient phenotype characterized by a larger decrease in EE during fasting and modest increases in EE during over-feeding [3,4]. Although the mechanisms underlying the metabolic EE response to dietary intervention are not fully elucidated, it has been suggested that changes in body composition [47], hormone levels [55,56,57,58], core body temperature [53] brown adipose tissue activity [59,60], and sympathetic nervous system tone [61,62,63] might play a role in the regulation of the “adaptive metabolic mechanism”.

“Metabolic adaption” is defined as a physiological mechanism leading to a reduction in RMR following weight loss, which is greater than that predicted by fat mass and fat free mass reduction [64]. This adaptation is thought to be one of the mechanisms whereby the body resists further weight loss, thus leading subjects to easily regain weight. Milestone studies have demonstrated a marked and persistent metabolic adaption following weight loss both in normal weight and obese subjects [65,66]. The degree of metabolic adaptation is related not only to the extent of weight loss, but also to the velocity of body weight reduction [67]. In subjects with obesity who participated in the televised weight-loss competition “Biggest Loser” and lost nearly 40% of their initial body weight during a 30 week-program thanks to a calorie-restricted diet greater than 70% of their baseline energy requirements and supervised vigorous training, a very remarkable metabolic adaption could be demonstrated, despite a relative preservation of their fat-free mass [67]. Fothergill et al. demonstrated that contestants of “The Biggest Loser” showed a metabolic adaption (of approximately 500 kcal/day) which persisted six years after the end of the TV-show and despite a marked weight regain [68]. In recent perspectives [64,69], it has been highlighted that a major reduction in body weight, such as the one obtained by the participants of the “Biggest Loser”, is almost impossible to be maintained when subjects return to their natural environment unless an extraordinarily high level of physical activity is kept. Major physiological responses to weight loss, which explain the weight regain phenomenon, are the persistence of a lower RMR, limited attitude towards physical activity, reduction in fat oxidation, and persistence of an increased appetite caused by the simultaneous increase of orexigenic and reduction of anorexic signals [70]. In particular, hormones, such as leptin, insulin, peptide YY (PYY), oxyntomodulin, irisin, adiponectin, and ghrelin, control food intake and energy balance by regulating orexigenic and anorexigenic pathways acting on the hypothalamic nuclei [49,71,72].

## 5. The Impact of Ketogenic Diet on Energy Expenditure

With this premise in mind, it is intriguing to ask which effects KD, a calorie-restricted dietary intervention for weight loss, may display on energy expenditure. Studies on this topic are unfortunately scant and somehow controversial. Furthermore, no studies have been conducted following patients in the long-term and possibly documenting the effects of KD on metabolic adaption. In the last decade, some clinical trials evaluating the role of KD on body weight and body composition have shown that patients assigned to a VLCKD and compared to individuals fed with a standard LC diet showed a higher reduction in body weight, waist circumference, and body fat mass with no measurable change in the fat free mass [27,73,74,75], suggesting a role of KD in sparing the lean mass.

In the study of Ebbeling et al. [76], 21 individuals with overweight or obesity, after reaching a 10–15% reduction of body weight with a run-in diet, were randomized by a cross-over trial to an isocaloric low-fat diet (60% of energy from carbohydrate, 20% from fat, 20% from protein; high glycemic load), low-glycemic index diet (40% from carbohydrate, 40% from fat, and 20% from protein; moderate glycemic load), and very low-carbohydrate diet (10% from carbohydrate, 60% from fat, and 30% from protein; low glycemic load) for a four-week-period. During isocaloric feeding following weight loss, TEE was about 300 kcal/day greater with the VLC diet compared to the LF diet. At variance, in the study by Hall et al. in males with overweight or obesity, fed initially with a high-carbohydrate high-sugar diet for four weeks followed by a KD of the same caloric intake with comparable protein amount, only a small increase in TEE (by ~100 kcal/day) in the first week of KD intervention was observed, followed by a linear decrease of TEE over time [77]. In that study, the increase of EE was observed during sleep, whereas no change in PA and TEF were observed. In a study conducted in women with obesity randomly assigned to a VLCKD or LF diet with similar caloric intake, although the reduction in fat mass and fat free mass was twice higher in those fed with VLCKD, no difference in REE measured by indirect calorimeter was observed [78]. In a randomized controlled trial [79], 32 individuals with overweight were divided into two groups. One underwent three dietary intervention steps (20-day period of LCKD with 848 kcal/day; 20-day period of LC non-ketogenic diet with 938 kcal/day; two-month period of Mediterranean diet (MD) with 1400 kcal/day), whereas the other group was assigned to a three-step regimen (20-day period of MD with 1200 kcal/day, 20-day period of MD with 1400 kcal/day, two-month period of MD with 1400 kcal/day). No difference in REE was observed between KD and MD. Similarly, in a recent study conducted in 16 male soccer players randomized to VLCKD or Western diet, despite a reduction in fat free mass, no change in REE measured with indirect calorimetry was observed between the two groups [80]. The most important clinical trials investigating the effect of KD on energy expenditure are reported in Table 1.

**Table 1:** Clinical trials investigating the effect of KD on energy expenditure.

Study	Study Design	Effect on Energy Expenditure
Ebbeling et al. [76]	21 individuals with overweight or obesity, after reaching a 10 to 15% reduction of body weight with a run-in diet, were randomized by a cross-over trial to an isocaloric low-fat diet (LF: 60% of energy from carbohydrate, 20% from fat, 20% from protein; high glycemic load), low-glycemic index diet (LGI: 40% from carbohydrate, 40% from fat, and 20% from protein; moderate glycemic load), and very low-carbohydrate diet (VLC:10% from carbohydrate, 60% from fat, and 30% from protein; low glycemic load) for a 4 week-period	TEE was about 300 kcal/day greater with the VLC diet compared to the LF diet. REE was 67 kcal/day higher with the VLC diet compared with the LF diet.
Hall et al. [77]	17 subjects with obesity fed with a high-carbohydrate baseline diet (BD) for 4 weeks followed by 4 weeks of an isocaloric KD with clamped protein	Small increase in TEE (by ~100 kcal/day) in the first week of KD intervention was observed, followed by a linear decrease of TEE over time. The increase of EE was observed during sleep whereas no change in PA and TEF were observed
Brehm [78]	50 women with obesity were randomized to 4 months of an ad libitum LC diet or an energy-restricted, low-fat diet	No difference in REE measured by indirect calorimeter was observed
Rubini [79]	32 healthy subjects were divided into 2 groups: one underwent 3 dietary intervention steps (20-day period of LCKD with 848 kcal/day; 20-day period of LC non-ketogenic diet with 938 kcal/day; 2-month period of Mediterranean diet (MD) with 1400 kcal/day) whereas the other group was assigned to a 3 step-regimen (20-day period of MD with 1200 kcal/day, 20-day period of MD with 1400 kcal/day, 2-month period of MD with 1400 kcal/day).	No difference in REE was observed between KD and MD
Paoli [80]	16 male soccer players randomized to VLCKD or Western Diet	No change in REE

TEE: total energy expenditure; REE: resting energy expenditure; TEF; thermogenic effect of food; PA: physical activity.

It has to be acknowledged that some of the studies reported in the literature may have significant limitations, which include different study design, lack of measurements of body composition, and imperfect measurements of energy expenditure and its components over the 24 h. These methodological differences do not allow a direct comparison between studies and definitive conclusions regarding the role of KD on energy metabolism.

Overall, it has been hypothesized [77] that during the first phase of KDs, when glucose utilization is still prevalent, an increase in EE may occur possibly due to increased hepatic oxygen consumption, proportional to the rate of ketogenesis and consequent to a raise in the energy request for gluconeogenic pathways and for triglyceride-fatty acid recycling [81,82,83]. Later on, a reduction of gluconeogenesis caused by the shifting from glucose to ketone bodies oxidation by the brain, a decrease in the respiratory quotient, and the possible direct action of additional hormonal signals (i.e., thyroid hormone, adipokines and catecholamines) might lead to a reduction in EE [84,85,86,87] (Figure 1). KDs might also act on long-lasting adaptations, by attenuating the decline of REE, which would occur following a reduction of body mass, but the lack of long-term follow-up studies in this regard does not allow us to speculate on this possibility.

## 6. Conclusions

Several lines of evidence support the efficacy of KDs as a strategy for fat loss. Studies so far reported do not show a clear EE response to extreme carbohydrate limitation. Minor changes in EE may occur in various phases of KD intervention due to the shift to different metabolic processes. A lack of long term follow up studies does not allow to shed light on the long-lasting adaptations (lower EE, reduced fat oxidation, changes in body composition, and greater appetite signals) to KD restrictions, which may occur following weight loss and might expose individuals to weight regain over time.

## Data Availability

Not applicable.

## Abbreviations

KD: ketogenic diet; LCD: Low-carbohydrate diet; VLCD: very-low-carbohydrate diets; LF: low fat diet; LCKD: low-calorie ketogenic diet; VLCKD: very low-calorie ketogenic diet; EI: energy intake; EE: energy expenditure; PA; physical activity; RMR: resting metabolic rate; TEF: thermic effect of food.
